# An Uncommon Case of Pyogenic Spondylodiscitis Caused by *Gemella morbillorum*

**DOI:** 10.1155/2018/3127613

**Published:** 2018-08-14

**Authors:** Takashi Sono, Mitsuru Takemoto, Koh Shinohara, Yasuhiro Tsuchido

**Affiliations:** ^1^Department of Orthopaedic Surgery, Kyoto City Hospital, Kyoto, Japan; ^2^Department of Infectious Diseases, Kyoto City Hospital, Kyoto, Japan; ^3^Department of Clinical Laboratory Medicine, Kyoto University Graduate School of Medicine, Kyoto, Japan

## Abstract

An 81-year-old man presented with severe back pain. Magnetic resonance imaging detected L5/S discitis without signs of epidural abscess. Punctures of the disc revealed that the causative organism was *Gemella morbillorum (G. morbillorum)*, which is part of the normal flora of the oral cavity and an uncommon causative pathogen of spondylodiscitis. The E-test method was useful for rapid susceptibility testing. Intravenous penicillin G treatment was effective, and the patient recovered without surgery.

## 1. Introduction

Pyogenic spondylitis is a comparatively rare disease, with incidence rates of 0.2 to 2.0 cases per 100,000 persons per year [1]. In Japan, the incidence increased to 7.4 cases per 100,000 persons per year in 2010 and the inhospital mortality rate was 6% [2]. Common causative organisms are *Staphylococcus aureus*, *Streptococcus* species, *Escherichia coli*, and *Proteus*. In immunocompromised patients, causative organisms included coagulase-negative *Staphylococcus* and viridans *Streptococcus* [1]. We report a case of spondylodiscitis caused by an uncommon pathogen, *Gemella morbillorum (G. morbillorum)*, and review the literature on this topic.

## 2. Case Presentation

**Figure 1:**
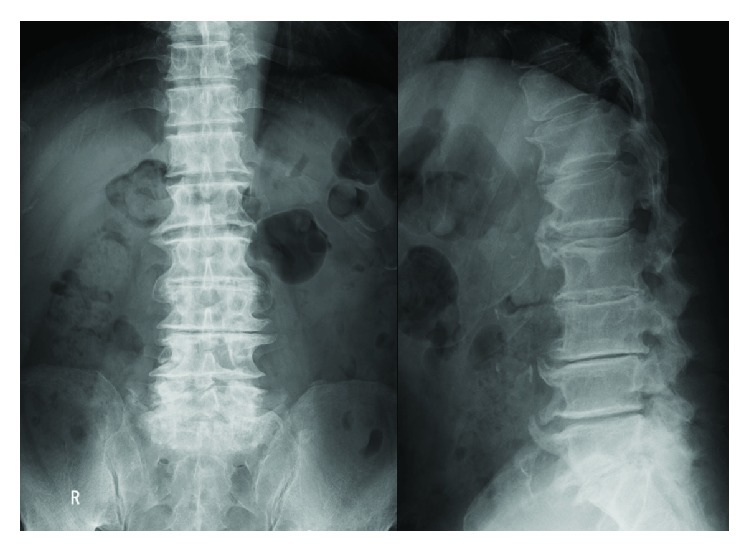
X-ray films of the lumbar spine. Severe spinal degeneration, but no signs of lumbar fracture, was observed.

**Figure 2:**
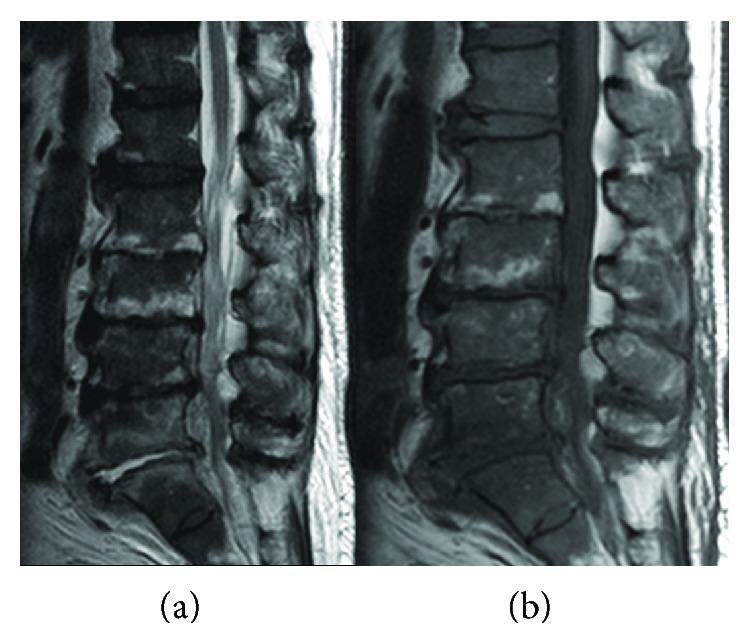
Magnetic resonance images of the lumbar spine. High-intensity lesion at the L5/S disc without epidural abscess was detected on a T2-weighted image (a). A low-intensity lesion at the same level was detected on a T1-weighted image (b).

The patient was an 81-year-old man with a history of left total hip replacement, open discectomy at the L4/5 level more than 10 years prior, percutaneous coronary intervention 3 years prior, and periodontitis detected 1 month before presentation. He suffered from severe back pain of 2-day duration. Plain lumbar spine radiographs showed spondylosis but no signs of fractures (Figure 1). Laboratory tests were significant for a white blood cell count of 1.2 × 10^4^ cells/*μ*l and C-reactive protein level of 13.8 mg/dl (Table 1). He was admitted for treatment. Two days after admission, magnetic resonance imaging of the lumbar spine revealed discitis at the L5/S level (Figure 2). Punctures of the disc were performed from both the left and right side under fluoroscopy, and two samples were obtained. Two sets of blood cultures and urine cultures were collected at the same time. Empiric therapy was started with vancomycin 1 g every 12 hours and ceftriaxone 1 g every 24 hours combined with lumbosacral orthosis. The culture of the disc aspirate was positive after 6 days, with the causative agent identified as *G. morbillorum* based on matrix-assisted laser desorption/ionization time-of-flight mass spectrometry analysis, performed with a Bruker Daltonics Microflex LT system (Bruker Daltonics, Germany). Blood and urine cultures were negative. Transthoracic echocardiogram showed no evidence of endocarditis. We could not perform the broth microdilution method for susceptibility testing because the isolate did not grow in the wells. Instead, we used the E-test method (SYSMEX bioMérieux) for determining susceptibility to penicillin G. Susceptibility of the isolate was interpreted by applying the Clinical and Laboratory Standards Institute (CLSI) M45-ED3. The minimum inhibitory concentration (MIC) of the isolate for penicillin G was 0.012 *μ*g/ml, which was interpreted as susceptible. Nine days from the initial treatment, antibiotic therapy was changed to ampicillin 2 g every 6 hours for 4 weeks. Then, oral amoxicillin was administered for 3 weeks. Lumbago resolved after 4 weeks of treatment. The patient was discharged from the hospital after 6 weeks of treatment. The isolate was referred to the Department of Clinical Laboratory Medicine, Kyoto University Graduate School of Medicine, for 16S ribosomal RNA sequence analysis. A BLAST search for the sequence in GenBank database gave 99.86% identity (1418/1420 bp) as *G. morbillorum* (GenBank accession number L14327).

## 3. Discussion

*G. morbillorum* is a catalase-negative, gram-positive coccus that is part of the normal flora of the oral cavity and gastrointestinal tract [3]. It rarely causes human infection but has been described previously in cases of endocarditis [4], meningitis [5], septic arthritis [6], and liver abscess [7].

To our knowledge, only five cases of spondylodiscitis caused by *G. morbillorum* have been reported, including the present case, as shown in Table 2. Since *G. morbillorum* is included in the normal flora of the oral cavity, dental injury and periodontitis are listed as predisposing symptoms. Infection is more common in the lumbar spine and in male patients. Broad spectrum cephem derivatives or carbapenems in combination with vancomycin are selected as empiric antibiotic treatments. The optimal duration of antibiotic treatment is 6 to 8 weeks [1]. Absolute surgical indications include spinal cord compression with progressive neurological deficits. In this situation, emergent posterior decompression should be considered. Relative surgical indications include minimal improvement with conservative treatment or progressive spinal deformity due to biomechanical instability [1]. Since spinal infections commonly affect the vertebral body, an anterior surgical approach is usually performed. By this approach, radical debridement of the infected site and placement of bone grafts or cages can be achieved. For lesions of the lumbar spine, a retroperitoneal or transperitoneal approach can be considered. Recently, surgery with anterior or posterior spinal instrumentation has been used as a treatment method to stabilize the affected spine [8, 9].

Although the majority of the *G. morbillorum* isolates from various clinical samples were reported to be susceptible to penicillin G and ampicillin, there are reported cases of infection with penicillin-resistant *G. morbillorum* isolates [10, 11]. The E-test on Brucella HK agar plates was used in this case. An inoculum with a fresh sterile cotton swab was applied to a 90 mm plate containing Brucella agar media, supplemented with 5% sterile defibrinated sheep blood, added to 600 *μ*l vitamin K_1_ and 600 *μ*l bovine hemin. The E-test strip containing penicillin G was applied to the plate. This test represents a simple and rapid method for quantitative susceptibility testing that is suitable for oral microorganisms [12]. The susceptibility was determined by the CLSI M45-ED3.

In conclusion, we experienced a rare case of spondylitis due to *G. morbillorum*. When a patient has a history of dental injury, periodontitis, or transplantation surgery, *G. morbillorum* can be a causative organism of spondylodiscitis. The E-test method is useful for the selection of antibiotics when bacterial growth is poor in broth media for microdilution testing.

## Figures and Tables

**Table 1:** Laboratory data.

RBC	340 × 10^4^/*μ*l
Hb	11.3 g/dl
Ht	33.40%
Plt	44.9 × 10^4^/*μ*l
WBC	12,030/*μ*l
CRP	13.77 mg/dl
TP	5.8 g/dl
ALB	2.6 g/dl
BUN	17.6 mg/dl
Cre	0.91 mg/dl
AST	96 U/l
ALT	96 U/l
ALP	329 U/l
Na	138 mEq/l
K	4.1 mEq/l
Cl	105 mEq/l

RBC: red blood cell; Hb: hemoglobin; Ht: hematocrit; Plt: platelet; WBC: white blood cell; CRP: C-reactive protein; TP: total protein; ALB: albumin; BUN: blood urea nitrogen; Cre: creatinine; AST: aspartate transaminase; ALT: alanine transaminase; ALP: alkaline phosphatase; Na: sodium ion; K: potassium ion; Cl: chloride ion.

**Table 2:** Characteristics of spondylodiscitis caused by *G. morbillorum*.

	Age, sex	Site	Predisposing factors	Epidural abscess	Antibiotic therapy	Surgery
Eisenberger et al. [13]	55, female	Thoracic	Renal transplantation, endocarditis	(+)	CTRX, CLDM, BZP	(−)
Nakayama et al. [14]	64, male	Cervical	Dental injury	(+)	ABPC/SBT, FMOX	(+)
Garcia-Bordes et al. [15]	53, male	Lumbar	None	(+)	IPM + VCM, CTRX	(+)
Hayasaka et al. [16]	54, male	Lumbar	None	(+)	MEPM, PIPC + MINO	(−)
Present case	81, male	Lumbar	Periodontitis	(−)	VCM + CTRX, PCG, AMPC	(−)

CTRX: ceftriaxone; CLDM: clindamycin; BZP: benzylpenicillin; ABPC/SBT: ampicillin/sulbactam; FMOX: flomoxef; IPM: imipenem; VCM: vancomycin; MEPM: meropenem; PIPC: piperacillin; MINO:minomycin; PCG: penicillin G; AMPC: amoxicillin.
